# 3,3′-Di-*tert*-butyl-5,5′-dimethoxy­biphenyl-2,2′-diol

**DOI:** 10.1107/S1600536809023071

**Published:** 2009-06-24

**Authors:** Zhong-Xiang Du, Ling-Zhi Wang

**Affiliations:** a Department of Chemistry, Luoyang Normal University, Luoyang, Henan 471022, People’s Republic of China; b Equipment Department, Luoyang Normal University, Luoyang, Henan 471022, People’s Republic of China

## Abstract

The title compound, C_22_H_30_O_4_, displays twofold rotational symmetry. The two benzene rings are almost perpendicular to each other, forming a dihedral angle of 89.8 (6)°. In the crystal, mol­ecules are linked into an extended one-dimensional chain structure *via* inter­molecular O—H⋯O hydrogen bonds.

## Related literature

For the various methods of preparing di-BHA [a dimer of 3-*tert*-butyl-4-hydroxy­anisole], see: Hewgill & Hewitt (1967 ▶); Jarl *et al.* (2004 ▶); Masahiro *et al.* (2005 ▶); Seiichiro *et al.* (2004 ▶).
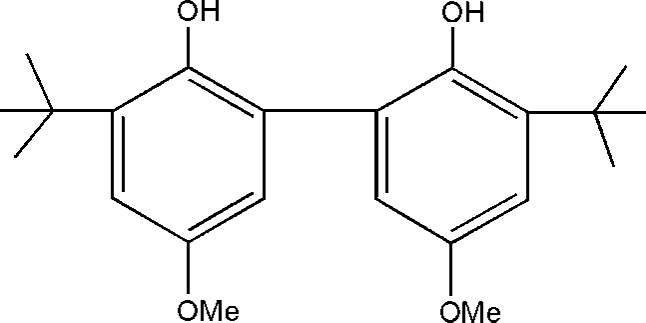


## Experimental

### 

#### Crystal data

C_22_H_30_O_4_
*M* *_r_* = 358.46
Tetragonal, 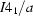

*a* = 13.4289 (8) Å
*c* = 23.127 (3) Å
*V* = 4170.5 (6) Å^3^
*Z* = 8
Mo *K*α radiation
μ = 0.08 mm^−1^
*T* = 291 K
0.49 × 0.49 × 0.38 mm

#### Data collection

Bruker APEXII CCD area-detector diffractometer
Absorption correction: multi-scan (*SADABS*; Sheldrick, 1996 ▶) *T*_min_ = 0.963, *T*_max_ = 0.972
13638 measured reflections
1938 independent reflections
1542 reflections with *I* > 2σ(*I*)
*R*_int_ = 0.025

#### Refinement

*R*[*F* ^2^ > 2σ(*F* ^2^)] = 0.041
*wR*(*F* ^2^) = 0.112
*S* = 1.04
1938 reflections
123 parameters
H-atom parameters constrained
Δρ_max_ = 0.14 e Å^−3^
Δρ_min_ = −0.13 e Å^−3^

### 

Data collection: *APEX2* (Bruker, 2004 ▶); cell refinement: *SAINT* (Bruker, 2004 ▶); data reduction: *SAINT*; program(s) used to solve structure: *SHELXS97* (Sheldrick, 2008 ▶); program(s) used to refine structure: *SHELXL97* (Sheldrick, 2008 ▶); molecular graphics: *SHELXTL* (Sheldrick, 2008 ▶); software used to prepare material for publication: *SHELXTL*.

## Supplementary Material

Crystal structure: contains datablocks global, I. DOI: 10.1107/S1600536809023071/at2819sup1.cif

Structure factors: contains datablocks I. DOI: 10.1107/S1600536809023071/at2819Isup2.hkl

Additional supplementary materials:  crystallographic information; 3D view; checkCIF report

## Figures and Tables

**Table 1:** Hydrogen-bond geometry (Å, °)

*D*—H⋯*A*	*D*—H	H⋯*A*	*D*⋯*A*	*D*—H⋯*A*
O2—H2⋯O1^i^	0.82	2.08	2.7592 (15)	140

Symmetry code: (i) 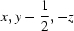.

**Fig. 1.**
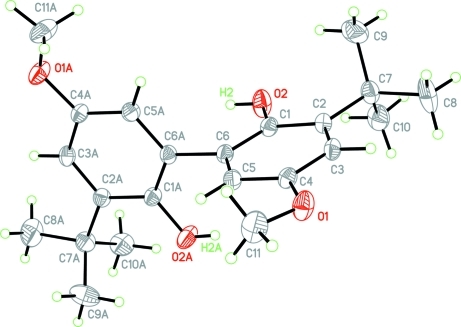
Molecular structure of (I), with displacement ellipsoids drawn at the 25% probability level. Atoms with suffix A are at the symmetry position (-x, -y + 3/2, z).

**Fig. 2.**
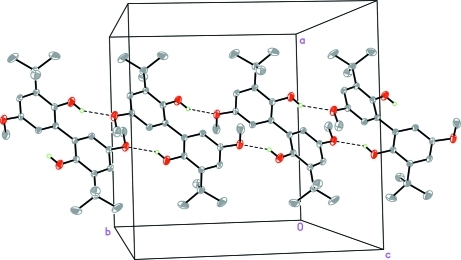
The crystal packing of (I), showing hydrogen bonds as dashed lines along b axis. H atoms on C atoms have been omitted.

## supplementary crystallographic information

### Comment

In the previous literatures, several methods for preparing di-BHA [a dimer of 3-*tert*-butyl-4-hydroxyanisole (BHA)] have been reported (Hewgill & Hewitt, 1967; Masahiro *et al.*, 2005; Jarl *et al.*, 2004; Seiichiro *et al.*, 2004), but its single-crystal and precise molecular structure has not been investigated so far. Here we describe the structure of the title compound, (I), (Fig. 1).

Di-BHA shows 2-fold rotational symmetry characters, where the 2-fold rotation axis is perpendicular to the C6—C6A bond. The oxygen atoms are almost coplanar with their own benzene ring-the largest deviation from the least-squares plane was found for O1 (or O1A), with an atom-plane distance of 0.017 Å. The two benzene rings have a dihedral angle of 89.8°, indicating that they are almost perpendicular to each other. The phenolic hydroxyl donor and methoxyl acceptor are involved in intermolecular hydrogen bonds and they extend di-BHA molecules into a one-dimensional chain structure along the *b* axis (Table 1, Fig.2), thus stabilizing di-BHA in the solid state.

### Experimental

An easy preparation method improved by Jarl *et al.* (2004) was adopted in our experiment. A solution of [K_3_Fe(CN)_6_] (0.1 mol, 3.29 g) and KOH (0.1 mol, 5.61 g) in water (100 ml) was prepared and was added dropwise to a solution of 3-*tert*-butyl-4-hydroxyanisole (0.1 mol, 1.80 g) in acetone (10 ml) over 3 h at room temperature. After vigorous agitation, yellow rice-shaped precipitate was obtained and filtered. Then the solid product was extracted with CH_2_Cl_2_ (3 × 50 ml), and the organic phase was dried over Na_2_SO_4_. After removal of CH_2_Cl_2_ under vacuum, a light brown solid was obtained. It turned into white crystal substance after washed with anhydrous ethanol(3 × 50 ml). Dissolve the white crystal substance in CH_2_Cl_2_ and filter the solution. About 4 days later, colourless block-shaped crystals suitable for X-ray diffraction analysis were appeared by slow evaporation in a yield of 63%. m. p. 510-511 K. Analysis, found: C 73.57, H 8.44%; C_22_H_30_O_4_ requires: C 73.65, H 8.37%. IR (KBr, ν, cm^-1^): 3412.6(ν O—H), 1594.2, 1455.6(ν (C_6_H_6_), skeleton), 1396.2, 1365.4(ν (CH_3_)_3_-C, skeleton), 1215.3, 1138.8(νC-O), 784.1(γ(C=C—H)).

### Refinement

H atoms bonded to C were positioned geometrically with C—H distance of 0.93–0.96 Å, and treated as riding atoms, with *U*_iso_(H)=1.2 or 1.5*U*_eq_(C). The O—H hydrogen atom was located in a difference Fourier map and the applied restraint of the O—H distance was 0.820 Å, with *U*_iso_(H)=1.5*U*_eq_(O).

### Crystal data

**Table:**

C_22_H_30_O_4_	*D*_x_ = 1.142 Mg m^−^^3^
*M**_r_* = 358.46	Mo *K*α radiation, λ = 0.71073 Å
Tetragonal, *I*4_1_/*a*	Cell parameters from 3994 reflections
Hall symbol: -I 4ad	θ = 3.0–25.5°
*a* = 13.4289 (8) Å	µ = 0.08 mm^−^^1^
*c* = 23.127 (3) Å	*T* = 291 K
*V* = 4170.5 (6) Å^3^	Block, colourless
*Z* = 8	0.49 × 0.49 × 0.38 mm
*F*(000) = 1552	

### Data collection

**Table:**

Bruker APEXII CCD area-detector diffractometer	1938 independent reflections
Radiation source: fine-focus sealed tube	1542 reflections with *I* > 2σ(*I*)
graphite	*R*_int_ = 0.025
φ and ω scans	θ_max_ = 25.5°, θ_min_ = 2.8°
Absorption correction: multi-scan (*SADABS*; Sheldrick, 1996)	*h* = −16→15
*T*_min_ = 0.963, *T*_max_ = 0.972	*k* = −15→16
13638 measured reflections	*l* = −28→27

### Refinement

**Table:**

Refinement on *F*^2^	Primary atom site location: structure-invariant direct methods
Least-squares matrix: full	Secondary atom site location: difference Fourier map
*R*[*F*^2^ > 2σ(*F*^2^)] = 0.041	Hydrogen site location: inferred from neighbouring sites
*wR*(*F*^2^) = 0.112	H-atom parameters constrained
*S* = 1.04	*w* = 1/[σ^2^(*F*_o_^2^) + (0.0476*P*)^2^ + 2.3626*P*] where *P* = (*F*_o_^2^ + 2*F*_c_^2^)/3
1938 reflections	(Δ/σ)_max_ < 0.001
123 parameters	Δρ_max_ = 0.14 e Å^−^^3^
0 restraints	Δρ_min_ = −0.13 e Å^−^^3^

### Special details

**Table:**

Geometry. All e.s.d.'s (except the e.s.d. in the dihedral angle between two l.s. planes)are estimated using the full covariance matrix. The cell e.s.d.'s are takeninto account individually in the estimation of e.s.d.'s in distances, anglesand torsion angles; correlations between e.s.d.'s in cell parameters are onlyused when they are defined by crystal symmetry. An approximate (isotropic)treatment of cell e.s.d.'s is used for estimating e.s.d.'s involving l.s. planes.
Refinement. Refinement of *F*^2^ against ALL reflections. The weighted *R*-factor *wR* and goodness of fit *S* are based on *F*^2^, conventional *R*-factors *R* are based on *F*, with *F* set to zero for negative *F*^2^. The threshold expression of *F*^2^ > σ(*F*^2^) is used only for calculating *R*-factors(gt) *etc*. and is not relevant to the choice of reflections for refinement. *R*-factors based on *F*^2^ are statistically about twice as large as those based on *F*, and *R*- factors based on ALL data will be even larger.

### Fractional atomic coordinates and isotropic or equivalent isotropic displacement parameters (Å^2^)

**Table:**

	*x*	*y*	*z*	*U*_iso_*/*U*_eq_	
C1	0.11953 (11)	0.79586 (10)	0.02439 (6)	0.0416 (4)	
C2	0.18758 (11)	0.87570 (10)	0.02195 (6)	0.0416 (4)	
C3	0.16557 (11)	0.95179 (11)	−0.01654 (6)	0.0431 (4)	
H3	0.2085	1.0060	−0.0188	0.052*	
C4	0.08231 (11)	0.95010 (10)	−0.05172 (6)	0.0408 (3)	
C5	0.01714 (11)	0.87114 (10)	−0.04926 (6)	0.0417 (4)	
H5	−0.0385	0.8696	−0.0732	0.050*	
C6	0.03503 (10)	0.79350 (10)	−0.01059 (6)	0.0387 (3)	
C7	0.28338 (12)	0.87786 (13)	0.05857 (7)	0.0546 (4)	
C8	0.34307 (17)	0.97382 (18)	0.04844 (11)	0.0950 (8)	
H8A	0.3018	1.0305	0.0566	0.142*	
H8B	0.3647	0.9764	0.0089	0.142*	
H8C	0.4001	0.9746	0.0735	0.142*	
C9	0.34957 (16)	0.78944 (19)	0.04258 (11)	0.0878 (7)	
H9A	0.4062	0.7876	0.0679	0.132*	
H9B	0.3718	0.7964	0.0033	0.132*	
H9C	0.3123	0.7288	0.0465	0.132*	
C10	0.25901 (15)	0.87337 (17)	0.12326 (8)	0.0725 (6)	
H10A	0.2230	0.9322	0.1342	0.109*	
H10B	0.3197	0.8695	0.1451	0.109*	
H10C	0.2190	0.8157	0.1310	0.109*	
C11	0.00097 (17)	1.02543 (14)	−0.13233 (9)	0.0733 (6)	
H11A	0.0167	0.9698	−0.1567	0.110*	
H11B	0.0028	1.0856	−0.1547	0.110*	
H11C	−0.0644	1.0168	−0.1163	0.110*	
O1	0.07070 (9)	1.03143 (8)	−0.08749 (5)	0.0586 (3)	
O2	0.13978 (10)	0.72001 (8)	0.06230 (6)	0.0665 (4)	
H2	0.0997	0.6747	0.0574	0.100*	

### Atomic displacement parameters (Å^2^)

**Table:**

	*U*^11^	*U*^22^	*U*^33^	*U*^12^	*U*^13^	*U*^23^
C1	0.0462 (8)	0.0351 (7)	0.0437 (8)	−0.0028 (6)	−0.0020 (6)	0.0010 (6)
C2	0.0421 (8)	0.0389 (8)	0.0438 (8)	−0.0058 (6)	−0.0027 (6)	−0.0030 (6)
C3	0.0453 (8)	0.0369 (8)	0.0470 (8)	−0.0119 (6)	0.0005 (7)	−0.0021 (6)
C4	0.0483 (8)	0.0313 (7)	0.0427 (8)	−0.0033 (6)	−0.0002 (6)	0.0021 (6)
C5	0.0409 (8)	0.0403 (8)	0.0440 (8)	−0.0042 (6)	−0.0047 (6)	−0.0015 (6)
C6	0.0393 (8)	0.0338 (7)	0.0428 (8)	−0.0048 (6)	0.0025 (6)	−0.0024 (6)
C7	0.0491 (9)	0.0582 (10)	0.0565 (10)	−0.0083 (8)	−0.0133 (8)	−0.0002 (8)
C8	0.0763 (14)	0.1044 (17)	0.1042 (18)	−0.0472 (13)	−0.0422 (13)	0.0238 (14)
C9	0.0574 (12)	0.1129 (18)	0.0930 (16)	0.0194 (12)	−0.0211 (11)	−0.0159 (14)
C10	0.0739 (13)	0.0843 (14)	0.0595 (11)	−0.0043 (10)	−0.0228 (10)	−0.0031 (10)
C11	0.0946 (15)	0.0568 (11)	0.0686 (12)	−0.0020 (10)	−0.0309 (11)	0.0131 (9)
O1	0.0791 (8)	0.0406 (6)	0.0562 (7)	−0.0134 (5)	−0.0178 (6)	0.0115 (5)
O2	0.0759 (9)	0.0465 (7)	0.0770 (9)	−0.0174 (6)	−0.0289 (7)	0.0210 (6)

### Geometric parameters (Å, °)

**Table:**

C1—O2	1.3711 (18)	C8—H8A	0.9600
C1—C6	1.394 (2)	C8—H8B	0.9600
C1—C2	1.410 (2)	C8—H8C	0.9600
C2—C3	1.387 (2)	C9—H9A	0.9600
C2—C7	1.541 (2)	C9—H9B	0.9600
C3—C4	1.383 (2)	C9—H9C	0.9600
C3—H3	0.9300	C10—H10A	0.9600
C4—C5	1.3761 (19)	C10—H10B	0.9600
C4—O1	1.3788 (17)	C10—H10C	0.9600
C5—C6	1.395 (2)	C11—O1	1.400 (2)
C5—H5	0.9300	C11—H11A	0.9600
C6—C6^i^	1.500 (3)	C11—H11B	0.9600
C7—C9	1.529 (3)	C11—H11C	0.9600
C7—C10	1.533 (3)	O2—H2	0.8200
C7—C8	1.535 (3)		
			
O2—C1—C6	121.03 (13)	C7—C8—H8B	109.5
O2—C1—C2	117.52 (13)	H8A—C8—H8B	109.5
C6—C1—C2	121.45 (13)	C7—C8—H8C	109.5
C3—C2—C1	116.61 (13)	H8A—C8—H8C	109.5
C3—C2—C7	121.12 (13)	H8B—C8—H8C	109.5
C1—C2—C7	122.25 (13)	C7—C9—H9A	109.5
C4—C3—C2	122.53 (13)	C7—C9—H9B	109.5
C4—C3—H3	118.7	H9A—C9—H9B	109.5
C2—C3—H3	118.7	C7—C9—H9C	109.5
C5—C4—O1	124.28 (13)	H9A—C9—H9C	109.5
C5—C4—C3	120.16 (13)	H9B—C9—H9C	109.5
O1—C4—C3	115.55 (12)	C7—C10—H10A	109.5
C4—C5—C6	119.54 (13)	C7—C10—H10B	109.5
C4—C5—H5	120.2	H10A—C10—H10B	109.5
C6—C5—H5	120.2	C7—C10—H10C	109.5
C1—C6—C5	119.70 (12)	H10A—C10—H10C	109.5
C1—C6—C6^i^	121.90 (13)	H10B—C10—H10C	109.5
C5—C6—C6^i^	118.31 (12)	O1—C11—H11A	109.5
C9—C7—C10	109.25 (17)	O1—C11—H11B	109.5
C9—C7—C8	108.15 (18)	H11A—C11—H11B	109.5
C10—C7—C8	107.07 (16)	O1—C11—H11C	109.5
C9—C7—C2	109.75 (14)	H11A—C11—H11C	109.5
C10—C7—C2	110.95 (14)	H11B—C11—H11C	109.5
C8—C7—C2	111.58 (14)	C4—O1—C11	118.33 (12)
C7—C8—H8A	109.5	C1—O2—H2	109.5
			
O2—C1—C2—C3	−179.81 (14)	O2—C1—C6—C6^i^	−2.8 (2)
C6—C1—C2—C3	0.6 (2)	C2—C1—C6—C6^i^	176.75 (13)
O2—C1—C2—C7	1.9 (2)	C4—C5—C6—C1	−1.1 (2)
C6—C1—C2—C7	−177.67 (14)	C4—C5—C6—C6^i^	−177.55 (13)
C1—C2—C3—C4	−1.0 (2)	C3—C2—C7—C9	−117.04 (18)
C7—C2—C3—C4	177.31 (14)	C1—C2—C7—C9	61.1 (2)
C2—C3—C4—C5	0.3 (2)	C3—C2—C7—C10	122.12 (17)
C2—C3—C4—O1	179.81 (14)	C1—C2—C7—C10	−59.7 (2)
O1—C4—C5—C6	−178.71 (14)	C3—C2—C7—C8	2.8 (2)
C3—C4—C5—C6	0.7 (2)	C1—C2—C7—C8	−179.02 (17)
O2—C1—C6—C5	−179.17 (14)	C5—C4—O1—C11	−13.7 (2)
C2—C1—C6—C5	0.4 (2)	C3—C4—O1—C11	166.78 (16)

Symmetry codes: (i) −*x*, −*y*+3/2, *z*.

### Hydrogen-bond geometry (Å, °)

**Table:**

*D*—H···*A*	*D*—H	H···*A*	*D*···*A*	*D*—H···*A*
O2—H2···O1^ii^	0.82	2.08	2.7592 (15)	140

Symmetry codes: (ii) *x*, *y*−1/2, −*z*.
